# Detection of Error-Related Potentials in Stroke Patients from EEG Using an Artificial Neural Network

**DOI:** 10.3390/s21186274

**Published:** 2021-09-18

**Authors:** Nayab Usama, Imran Khan Niazi, Kim Dremstrup, Mads Jochumsen

**Affiliations:** 1 Department of Health Science and Technology, Aalborg University, 9000 Aalborg, Denmark; nu@hst.aau.dk (N.U.); kdn@hst.aau.dk (K.D.); mj@hst.aau.dk (M.J.); 2 Centre for Chiropractic Research, New Zealand College of Chiropractic, Auckland 1060, New Zealand; 3 Health and Rehabilitation Research Institute, AUT University, Auckland 0627, New Zealand

**Keywords:** error-related potentials, brain–computer interface, calibration, neurorehabilitation, stroke, classifier interpretation

## Abstract

Error-related potentials (ErrPs) have been proposed as a means for improving brain–computer interface (BCI) performance by either correcting an incorrect action performed by the BCI or label data for continuous adaptation of the BCI to improve the performance. The latter approach could be relevant within stroke rehabilitation where BCI calibration time could be minimized by using a generalized classifier that is continuously being individualized throughout the rehabilitation session. This may be achieved if data are correctly labelled. Therefore, the aims of this study were: (1) classify single-trial ErrPs produced by individuals with stroke, (2) investigate test–retest reliability, and (3) compare different classifier calibration schemes with different classification methods (artificial neural network, ANN, and linear discriminant analysis, LDA) with waveform features as input for meaningful physiological interpretability. Twenty-five individuals with stroke operated a sham BCI on two separate days where they attempted to perform a movement after which they received feedback (error/correct) while continuous EEG was recorded. The EEG was divided into epochs: ErrPs and NonErrPs. The epochs were classified with a multi-layer perceptron ANN based on temporal features or the entire epoch. Additionally, the features were classified with shrinkage LDA. The features were waveforms of the ErrPs and NonErrPs from the sensorimotor cortex to improve the explainability and interpretation of the output of the classifiers. Three calibration schemes were tested: within-day, between-day, and across-participant. Using within-day calibration, 90% of the data were correctly classified with the entire epoch as input to the ANN; it decreased to 86% and 69% when using temporal features as input to ANN and LDA, respectively. There was poor test–retest reliability between the two days, and the other calibration schemes led to accuracies in the range of 63–72% with LDA performing the best. There was no association between the individuals’ impairment level and classification accuracies. The results show that ErrPs can be classified in individuals with stroke, but that user- and session-specific calibration is needed for optimal ErrP decoding with this approach. The use of ErrP/NonErrP waveform features makes it possible to have a physiological meaningful interpretation of the output of the classifiers. The results may have implications for labelling data continuously in BCIs for stroke rehabilitation and thus potentially improve the BCI performance.

## 1. Introduction

A brain–computer interface (BCI) is a means for people with motor impairments to control external devices using only brain activity [1]. Individuals with severe motor impairments can use it for controlling, e.g., wheelchairs, spelling devices, and for inducing neural plasticity in stroke patients during motor rehabilitation [2,3]. A BCI typically consists of different building blocks: signal acquisition, pre-processing, feature extraction, classification, and device commands, and several advances have been made over the years to improve each of the building blocks. For optimal control of the BCI, the classifiers need to be calibrated to be individualized for the user to account for the non-stationarity of the EEG, but it takes time to collect data for calibrating them. Different approaches have been proposed in the literature to use a generalized classifier where the BCI works without the need of individualized training data [4,5,6,7]. However, user-specific classifiers based on within-day calibration data generally perform better, but they also suffer from changes in the brain activity that cause an inadequate representation with respect to the distribution of brain activity in the calibration data. These changes may be due to shifts in attention and fatigue, which may be pronounced in people with neurological diseases such as stroke [8]. A way to overcome this and optimize the performance of a BCI could be through the detection of error-related potentials (ErrPs), where the detection of this signal can be used for error correction or labelling data [9]. Error correction and labelling of data would work in different scenarios, e.g., error correction can be used for automatically deleting mistyped letters in a speller application or reverting the movement of a robotic arm, i.e., the application would be applicable for communication and control purposes. In BCI-based stroke rehabilitation (and the aforementioned applications as well), data labelling would also be applied either for updating a classifier continuously to reduce the effect of fatigue during a rehabilitation session. Moreover, it could be used to transfer from a generalized classifier that could be used initially to avoid collecting user-specific training data for the BCI before the actual rehabilitation training starts to an individualized classifier. It has been shown in several studies that ErrPs can be decoded (see [9,10] for a review about decoding ErrPs) and how the performance of the decoder can be optimized using different types of features [11,12,13,14,15,16,17,18,19,20,21], classifiers [11,12,17,18,22,23], time windows [24,25,26], and channels [17,25,27,28]. Moreover, it has been shown that generalized ErrP detectors can be transferred across different tasks and types of ErrPs such as observation and interaction ErrPs, across participants, and time which eliminates the need for calibration (see, e.g., [11,29,30,31,32,33,34,35,36]). This transfer may be successful due to the stability of the ErrP over time as good test/retest reliability has been reported for evoking the ErrP [37,38,39]. However, these studies have been conducted with healthy participants. This applies to the majority of the ErrP decoding literature as well with some exceptions. A few studies have investigated the decoding of ErrPs in people with motor impairments after amyotrophic lateral sclerosis [27] and spinal cord injury [14,31,40] but the literature for decoding of ErrPs within stroke is scarce. Studies are needed to address this, especially since a growing body of research has emerged within BCI-based rehabilitation after stroke [2]. As outlined previously, in this application ErrPs could be used to maintain good BCI performance by alleviating the effect of fatigue. Some studies have shown that ErrPs can be elicited in people with stroke [41,42,43], brain lesions [44,45,46,47,48,49], and traumatic brain injuries [50], but there is some variability in the ErrP morphology known from healthy volunteers. Generally, this is manifested through smaller or attenuated peak amplitudes. Despite the literature about eliciting ErrPs in stroke, there are no studies investigating the decoding of ErrPs. Additionally, with the variability of ErrP morphology, it may be difficult to make a generalized ErrP detector across people with stroke and across days. The aim of this study was threefold: (1) decode ErrPs in people with stroke using different calibration schemes, within-day, between-day, and across-participant, (2) investigate the reliability of the decoder across two separate days, and (3) compare different classification methods. These classification problems were assessed with waveform features as input to the classifier from the sensorimotor cortex to allow a meaningful physiological interpretability of the classifiers.

## 2. Methods

### 2.1. Participants

In this study, 25 stroke participants (see Table 1) were recruited. The experiments were conducted at Allied Hospital Faisalabad, Pakistan. All participants provided their written consent before the experiment, and the local ethical committee at Allied Hospital approved the study. The stroke participants were recruited through the Department of Neurology at Allied Hospital Faisalabad. Prior to the experiments, the impairment level of the stroke participant’s affected limb was assessed by a neurologist. The impairment level was quantified using the Brunnstrom Stage classification [51]. Additionally, eight able-bodied participants (3 females; age = 42 ± 11 years) were included in the Supplementary Materials.

### 2.2. Data Recording

**Figure 1:**
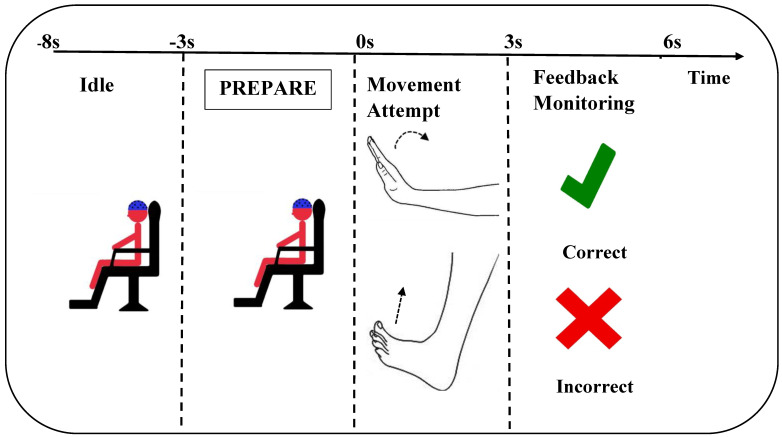
Timeline of a single trial. The participant is cued to perform a specific movement after which the movement is attempted immediately after the presentation of the cue. The sham feedback was provided to the participant three seconds after the attempted movement.

64 channels EEG were recorded with a sampling rate of 1200 Hz using active electrodes (g.HIamp G.Tec, Graz, Austria). The electrodes were placed according to the 10-10 system. A linked ear reference was used, and the ground electrode was located at AFz. The impedance of all electrodes was kept below 5 kΩ during the experiments. By using an Arduino microcontroller, an external trigger was sent to the EEG amplifier from a custom-made MATLAB (MathWorks, 2018) interface software. The trigger pulses were recorded to synchronize the continuous EEG with the presentation of feedback for the participant and to divide the EEG into ErrP (‘Incorrect’ in Figure 1) and NonErrP (‘Correct’ in Figure 1) epochs.

### 2.3. Experimental Details

The participants performed the experiment while seated in a comfortable chair facing a computer screen where cues were shown to them throughout the experiment. Figure 1 shows the timeline for a single trial during the experiments. Each repetition of a trial started with an idle phase of five seconds, during which the participants could blink and relax. Subsequently, a preparation phase was started during which a text was displayed on the screen: ‘*Prepare for the movement*’. The preparation phase lasted for three seconds. Next, a picture of a hand or foot (pointing towards the right or left direction) was shown in the center of the computer screen to indicate movement of the respective hand (wrist extension) or foot (dorsiflexion). During the attempted movement and feedback monitoring phase, the participants were asked to avoid making eye movements by focusing their gaze on the screen, blinking, activate facial muscles and sit as still as possible. An equal number of repetitions was performed for each movement type, which was randomized for each participant individually. The participants were asked to attempt to execute the movement as soon as they saw the picture on the screen. Random feedback with a ratio of 70/30 (correct/error) was provided with a delay of three seconds in the form of a green tick mark or a red-colored cross sign as it has been shown to elicit ErrPs [18] to ensure that enough ErrPs and NonErrPs were elicited for the classification analyses within the time frame of the experiment. Before the experiment, it was conveyed to the participants that the system was decoding the intended movement from their brain signals, and the feedback type solely depended on their brain signals during the attempted movement [18]. 100 attempted movements were performed of each movement type, i.e., 400 attempted movements in total. The experiment was completed in 20 runs, where each run consisted of 20 trials. After the completion of each run, a break was given to the participants until they were ready to start the experiment again. The experiments were completed in approximately 150–180 min. The same experiment was performed on two separate days with a gap of approximately one month between the days. The brain activity during the presentation of correct or erroneous feedback was used for discrimination between NonErrPs and ErrPs.

### 2.4. Signal Processing

#### 2.4.1. Pre-Processing

Thirty-seven channels of EEG (AF3-4, Fz, F1-6, FCz, FC1-6, Cz, C1-6, CPz, CP1-6, Pz, P1-6) were used for the analysis. Initially, the channels were bandpass filtered between 0.05–10 Hz using an 8th order zero phase-shift Butterworth filter [9]. Afterwards, bad channels were excluded from further analysis for each participant individually. Bad channels were defined as having a mean amplitude more than three standard deviations above the overall mean amplitude of the 37 channels. The filtered data were divided into 400-millisecond epochs starting from 100 milliseconds after the time the feedback was presented (t = 3 s in Figure 1) and ending 500 milliseconds after the feedback was presented. Bad epochs were rejected from further analysis if they had amplitudes exceeding 150 µV. All trials were normalized by subtracting the mean value of the entire trial from each value in the trial. The number of ErrPs and NonErrPs was balanced. NonErrPs were randomly selected to match the number of included ErrP epochs in the remaining analyses.

#### 2.4.2. Classification

Two types of classifiers were used. A multi-layer perceptron artificial neural network (MLP ANN) and shrinkage linear discriminant analysis (LDA) were employed for the classification of ErrPs and NonErrPs for both recording days of the stroke and able-bodied participants. The classification with MLP ANN was performed with temporal features as input and with the entire epochs as input. The temporal features were the waveform signal values of the epochs downsampled to 50 Hz. The classification with ANN was compared with classification of the temporal features with LDA, which is a traditional approach for decoding ErrPs [9]. The waveform features from the channels over the sensorimotor cortex were chosen to capture the ErrP to allow interpretability of the output of the classifier.

A 5-layer MLP ANN was used [28,52] with an input layer equal to the number of features (number of channels x number of samples in epoch), three hidden layers of the size 100-50-25, and an output layer of size 1 with a sigmoid activation function. The scaled conjugate gradient descent method was used for training the neural network with a maximum number of epochs of 200. Network performance was checked by the cross-entropy, and to avoid the early stopping the validation checks were set equal to 200. Three different types of calibration were performed: (1) Within-day calibration was performed on the data for each participant individually using 10-fold cross-validation, (2) Between-day calibration was performed for each participant individually by calibrating the classifier on data from one day and testing on the other, and (3) Across-participant calibration was performed using leave-one-participant-out cross-validation, where the data from one participant were used for testing while the data from the other participants were used for training (the analysis was performed separately for stroke and able-bodied participants). All analyses were performed using MATLAB (MathWorks^®^).

### 2.5. Statistics

A two-way repeated measure analysis of variance (ANOVA) test was performed on the classification accuracies calibration schemes (3 levels: within-day, between-day, and across-participant) and classification method (3 levels: features classified with ANN, entire epoch classified with ANN, and features classified with sLDA) as factors. The mean of the classification accuracies obtained on the two days for each participant was used in the analysis. Significant test statistics were followed up with post hoc analysis using a Bonferroni correction to avoid multiple comparisons. The Greenhouse-Geisser correction was applied if the assumption of sphericity was violated. The reliability of the classification accuracies obtained for the within-day calibration scheme across the two days was tested using Pearson’s correlation. Lastly, Spearman’s correlation test was performed between the classification accuracies for the within-day calibration scheme (recording day one) and the Brunnstrom Stage score (obtained prior to recording day one). Statistical significance was assumed when *p* < 0.05 for all tests. The statistical analyses were performed using IBM^®^ SPSS^®^.

## 3. Results

**Figure 2:**
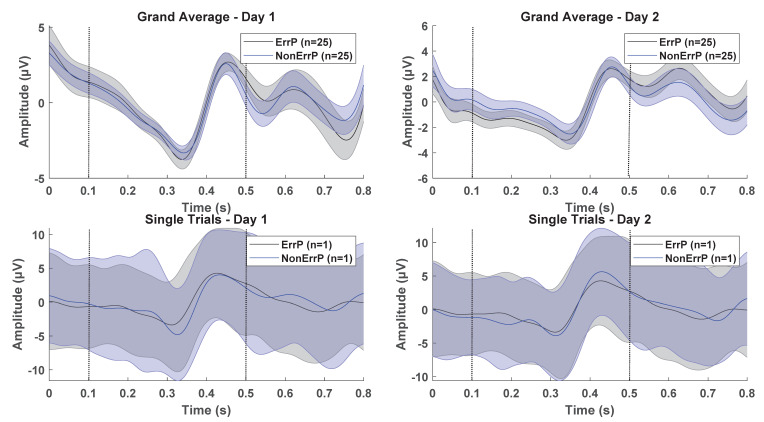
Top: Grand average across 25 participants for the ErrP and NonErrP epochs for recording day 1 and 2. The shaded area indicates the standard error across participants and the solid line is the mean. Bottom: Single trials for participant 1 for recording day 1 and 2. The shaded area indicates the standard deviation, and the solid line is the mean across the trials. Time ‘0 s’ is the onset of the presentation of the feedback. The signals in all trials are from the electrode position FCz. The vertical dotted lines indicate the part of the signal that was used for the classification analyses.

On average, 0.28 ± 0.50 channels (range: 0–1, maximum one channel was removed) and 72 ± 64 epochs (range: 2–228) were excluded for the stroke participants in the first recording day, and 0.32 ± 0.50 channels (range: 0–1) and 73 ± 75 epochs (range: 3–293) were excluded from the second recording day. A grand average across participants is presented in Figure 2 (see Figure S1 in Supplementary Materials for able-bodied participants) as well as a plot of the mean and standard deviation of single trials for one participant. For the grand averages, a negative peak is observed approximately 350 milliseconds after the presentation of the feedback and a positive peak 450 milliseconds after the presentation of the feedback. The ErrP and NonErrP epochs overlie with a slightly higher amplitude of the negative peak for the ErrPs compared with the NonErrPs. The shape of the ErrPs and NonErrPs are similar across the two recording days. For the single trials the ErrPs and NonErrPs overlap with a similar shape for both recording days.

**Figure 3:**
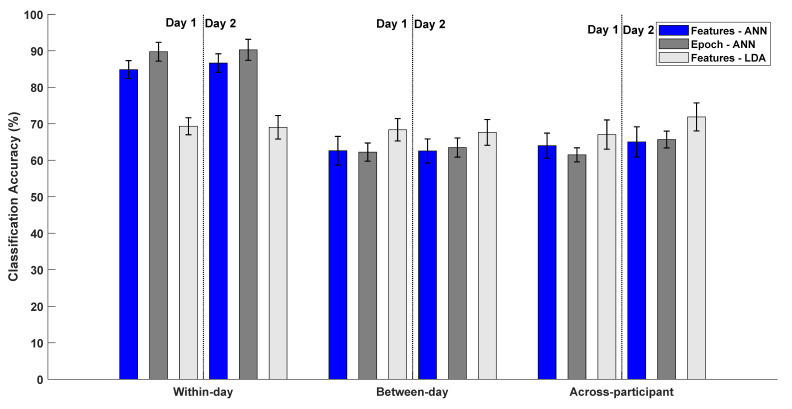
Classification accuracies associated with different calibration schemes using features or the entire epoch as input for the artificial neural network and classification of features using linear discriminant analysis. The bars represent the mean ± standard error across the participants. For the between-day calibration, the three bars under “Day 1” represent training the classifier on data from recording day 1 and testing on recording day 2 and vice versa for the three bars under “Day 2”.

The classification accuracies associated with the three different calibration schemes are summarized in Figure 3. The results in the following are presented as mean ± standard error.

The average within-day classification accuracies (see Figure 3) using features as input for the ANN were 84.9 ± 2.5% (day 1) and 86.7 ± 2.5% (day 2). When using the entire epoch as input for the ANN the within-day classification accuracies were 89.8 ± 2.5% (day 1) and 90.2 ± 2.9% (day 2). Lastly, the within-day classification accuracies with features and LDA were 69.3 ± 2.3% (day 1) and 69.0 ± 3.2% (day 2). There was no correlation between the classification accuracies obtained for the two recording days using features (Pearson’s correlation = 0.09; *p* = 0.66) or the epoch (Pearson’s correlation = −0.17; *p* = 0.43) with ANN or with features and LDA (Pearson’s correlation = −0.04; *p* = 0.85). The correlation analysis between the within-day classification accuracies of recording day 1 and the Brunnstrom Stage score revealed no association for ANN with features as input (Spearman’s correlation = 0.03; *p* = 0.88), ANN with epochs as input (Spearman’s correlation = 0.14; *p* = 0.52), and LDA with features as input (Spearman’s correlation = −0.01; *p* = 0.96).

The average between-day classification accuracies (see Figure 3) using features as input for ANN were 62.7 ± 3.9% (training on day 1 and testing on day 2) and 62.6 ± 3.3% (training on day 2 and testing on day 1). When using the entire epoch as input for ANN the between-day classification accuracies were 62.3 ± 2.5% (training on day 1 and testing on day 2) and 63.4 ± 2.6% (training on day 2 and testing on day 1). Lastly, when using features as input for LDA the between-day classification accuracies were 68.3 ± 3.1% (training on day 1 and testing on day 2) and 67.7 ± 3.5% (training on day 2 and testing on day 1).

The average across-participant classification accuracies (see Figure 3) using features as input for ANN were 64.0 ± 3.4% (day 1) and 65.2 ± 4.1% (day 2). When using the entire epoch as input for the ANN, the across-participant classification accuracies were 61.4 ± 1.9% (day 1) and 65.7 ± 2.3% (day 2). The average across-participant classification accuracies were 67.0 ± 4.0% (day 1) and 71.9 ± 3.8% (day 2) when using features as input for the LDA. The classification accuracies for the able-bodied participants for the within-day, between-day and across-participant calibration schemes are presented in Figure S2 in the Supplementary Materials.

The statistical analyses revealed a significant interaction between calibration scheme and classification method (F_(4,96)_ = 30.74; *p* < 0.001; η^2^ = 0.56), and a significant effect of calibration scheme (F_(2,48)_ = 37.73; *p* < 0.001; η^2^ = 0.61). The effect of the classification method was not significant (F_(2,48)_ = 1.86; *p* = 0.17; η^2^ = 0.07). The post hoc analysis revealed that significantly higher classification accuracies were obtained for within-day calibration compared with the other calibration schemes. Three one-way repeated measures ANOVA tests were performed with classification method as the factor for each calibration scheme individually. The results revealed a significant effect of within-day (F_(1.5,35.9)_ = 62.25; *p* < 0.001; η^2^ = 0.72) and across-participant calibration (F_(2,48)_ = 3.32; *p* = 0.04; η^2^ = 0.12), the effect of between-day calibration was not significant (F_(2,48)_ = 2.43; *p* = 0.10; η^2^ = 0.09). The post hoc test revealed that the ANN with features and epochs as input achieved higher classification accuracies than the LDA with features as input. In the across-participant calibration scheme, the LDA performed better than the ANN, but due to the conservative nature of the Bonferroni correction there was no significant difference (*p* = 0.06). 

## 4. Discussion

In this study it was shown that ErrPs could be classified with accuracies in the range of 90% using within-day calibration and the entire epoch as input for the ANN. The accuracies decreased to 86% and 69% when using temporal features as input for the ANN and LDA, respectively. The classification accuracies associated with between-day and across-participant calibration were significantly lower compared with the within-day calibration. There was poor reliability between the classification accuracies obtained on day 1 and 2, and there was no correlation between the classification accuracies and the impairment level of the participants. The classification accuracies for the different calibration schemes were significantly higher than chance level (calculated with a significance level of 5% [53]). The accuracies above chance level for the between-day and across-participant calibration schemes are in agreement with several other studies that have reported that ErrPs can be detected using these approaches [17,22,30,31,32,33,35,36,54]. The classification accuracies obtained for LDA in between-day and across-participant calibration are similar to what has been reported previously, but lower for ANN. This may suggest that better generalization across days and participants can be obtained with LDA compared with ANN. The grand averages presented in this study indicate a similar morphology on both days, and across participants there are only slight differences in amplitudes for the ErrPs and NonErrPs which makes it difficult to discriminate between them. The correlation analysis also showed there was poor reliability between the classification accuracies obtained on the two days, despite relatively high within-day classification accuracies, possibly due to variability across days. The findings in this study suggest that for people with stroke, using these classification approaches, it is necessary to train an ErrP detector on data from the same day, but it takes time to collect calibration data for this. In this study 400 trials with NonErrPs and ErrPs (70/30 ratio) were elicited and ~70 epochs of these were rejected. This number of NonErrPs and ErrPs was enough for calibrating a classifier to obtain good performance, but other approaches could be used to reduce the time it takes to collect calibration data such as changing the NonErrP/ErrP ratio to allow more ErrPs [54,55] or use observation ErrPs that are fast to elicit and that can generalize to other types of ErrPs such as interaction ErrPs [35]. Moreover, it may not be needed to use as many ErrP and NonErrP epochs as were utilized in this study; it has been reported that a steep increase in classification accuracies was observed after ~50–100 ErrPs were used for calibration [33,36,40]. The classification accuracies based on within-day calibration are in agreement with findings in several other studies which have reported a detection performance in the range of roughly 70–90% [12,13,15,16,17,22,28,30,32,33,56]; it should be noted that different classification metrics have been reported. This was the case when using either features or the entire epoch as input for the ANN classifier. The similar findings obtained for the two approaches for ANN were expected since waveform data were used in both cases (with/without downsampling). The fact that waveform data can be used indicates that identifying various feature types may not be needed to obtain good ErrP detection performance. Such features make it possible to make a meaningful physiological interpretation of the output of the classifier. It is also a challenging task to identify features that generalize well across time, such as individuals and ErrP paradigms, due to factors that affect the ErrP morphology such as temporal variability and jitter, although methods have been proposed for overcoming this [11,34]. By using the waveform data or epoch from the specific user, it is also possible to eliminate some of the factors that affect the ErrP morphology. Age has consistently been reported to reduce the ErrP amplitude (see, e.g., [57]), and factors such as the perceived severity of the error [16], awareness of error [41,58], and error type modulate the ErrP morphology [16,59,60]. The latter factors in particular may be more pronounced in individuals with stroke compared with able-bodied individuals. Many stroke patients may have some degree of cognitive impairment that may alter the awareness of an error, depending on how it was elicited. This could lead to lower ErrP peak amplitudes. On the contrary, stroke patients may have a stronger perception of error severity, leading to higher peak amplitudes. However, this will also be affected if they think it was the system and not themselves making an error, which will lead to smaller peak amplitudes. These are some questions that should be addressed in future studies where it would be relevant to include some qualitative data to better understand how these factors modulate the ErrP in a stroke population [41]. Moreover, future studies should also address online decoding of ErrPs in stroke patients when operating a real-time BCI in a relevant BCI user scenario such as rehabilitation with BCI-triggered electrical stimulation.

## 5. Conclusions

In conclusion, it was shown that ErrPs and NonErrPs in stroke patients could be classified correctly with high classification accuracies when using a within-day calibration scheme, but with poor test–retest reliability this was across days. The classification accuracies decreased significantly when applying between-day and across-participant calibration schemes. The best performance was obtained when using the entire ErrP and NonErrP input for the ANN classifier compared with temporal features classified with LDA. By using physiologically meaningful brain potentials (ErrP and NonErrP) as input for the classifiers, it may be possible to interpret the output with respect to the existing physiological research within this area. The results may have implications for using ErrP classification as a tool for labelling EEG data during BCI use in a stroke rehabilitation scenario. 

## Figures and Tables

**Table 1:** Stroke participant ID, gender, age (years), affected side due to stroke (hemiplegia), type of stroke, number of days since injury and Brunnstrom Stage (I = flaccidity, II = spasticity appearance, III = increased spasticity, IV = decreased spasticity, V = complex movement combinations, VI = spasticity disappears, VII = normal function returns) are shown.

Subject ID	Gender	Age (Years)	Affected Side	Type of Stroke	Time Since Injury (Days)	Brunnstrom Stage
1	M	48	Right	Haemorrhage	91	II
2	M	55	Right	Ischemic	172	V
3	M	41	Left	Ischemic	70	III
4	M	50	Left	Haemorrhage	90	III
5	M	57	Right	Haemorrhage	52	V
6	M	52	Right	Ischemic	188	V
7	M	24	Left	Haemorrhage	180	IV
8	F	32	Left	Ischemic	25	II
9	F	26	Left	Haemorrhage	20	I
10	M	60	Right	Ischemic	87	IV
11	M	54	Left	Ischemic	220	VII
12	M	46	Left	Ischemic	42	III
13	M	58	Right	Ischemic	84	III
14	M	37	Right	Haemorrhage	36	II
15	M	42	Left	Haemorrhage	118	V
16	M	24	Left	Haemorrhage	45	IV
17	F	26	Right	Ischemic	12	I
18	M	62	Right	Haemorrhage	118	III
19	M	30	Right	Ischemic	60	III
20	F	53	Left	Ischemic	93	IV
21	F	38	Right	Haemorrhage	45	VI
22	F	28	Left	Ischemic	27	V
23	M	45	Left	Ischemic	90	IV
24	M	35	Left	Haemorrhage	17	II
25	M	45	Right	Haemorrhage	280	VI

## Data Availability

The data is not publicly available because of ethics and institutional policy.

## Supplementary Materials

The following are available online at https://www.mdpi.com/article/10.3390/s21186274/s1, Figure S1: Top: Grand average across eight able-bodied participants for the ErrP and NonErrP epochs for recording day 1 and 2. The shaded area indicates the standard error across participants and the solid line is the mean. Bottom: Single trials for participant 1 for recording day 1 and 2. The shaded area indicates the standard deviation and the solid line is the mean across the trials. Time ‘0 s’ is the onset of the presentation of the feedback. The signals in all trials are from the electrode position FCz. The vertical dotted lines indicate the part of the signal that was used for the classification analyses. Figure S2: Classification accuracies associated with different calibration schemes using features or the entire epoch as input for the artificial neural network and classification of features using linear discriminant analysis. The bars represent the mean ± standard error across the able-bodied participants. For the between-day calibration, the three bars under “Day 1” represent training the classifier on data from recording day 1 and testing on recording day 2 and vice versa for the three bars under “Day 2”.
