# Supplementary material for: Detection of Error-Related Potentials in Stroke Patients from EEG Using an Artificial Neural Network
Source: Sensors (Basel). 2021 Sep 18;21(18):6274. doi: 10.3390/s21186274 (PMC8472485; doi:10.3390/s21186274)
Supplement: Supplementary file 1 [file sensors-21-06274-s001.zip › sensors-1352506-supplementary.pdf]

sensors

MDPI

Article

Detection of Error-Related Potentials in Stroke Patients from EEG Using an Artificial Neural Network

Nayab Usama [1], Imran Khan Niazi [1,2,3,*], Kim Dremstrup [1] and Mads Jochumsen [1]

[1] Department of Health Science and Technology, Aalborg University, Aalborg 9000, Denmark; nu@hst.aau.dk (N.U.); kdn@hst.aau.dk (K.D.); mj@hst.aau.dk (M.J.)
[2] Centre for Chiropractic Research, New Zealand College of Chiropractic, , Auckland 1060, New Zealand; imran.niazi@nzchiro.co.nz(I.K.N.)
[3] Health and Rehabilitation Research Institute, AUT University, Auckland 0627, New Zealand
* Correspondence: imran.niazi@nzchiro.co.nz; Tel.: +64-9-526-6789

Supplementary Material

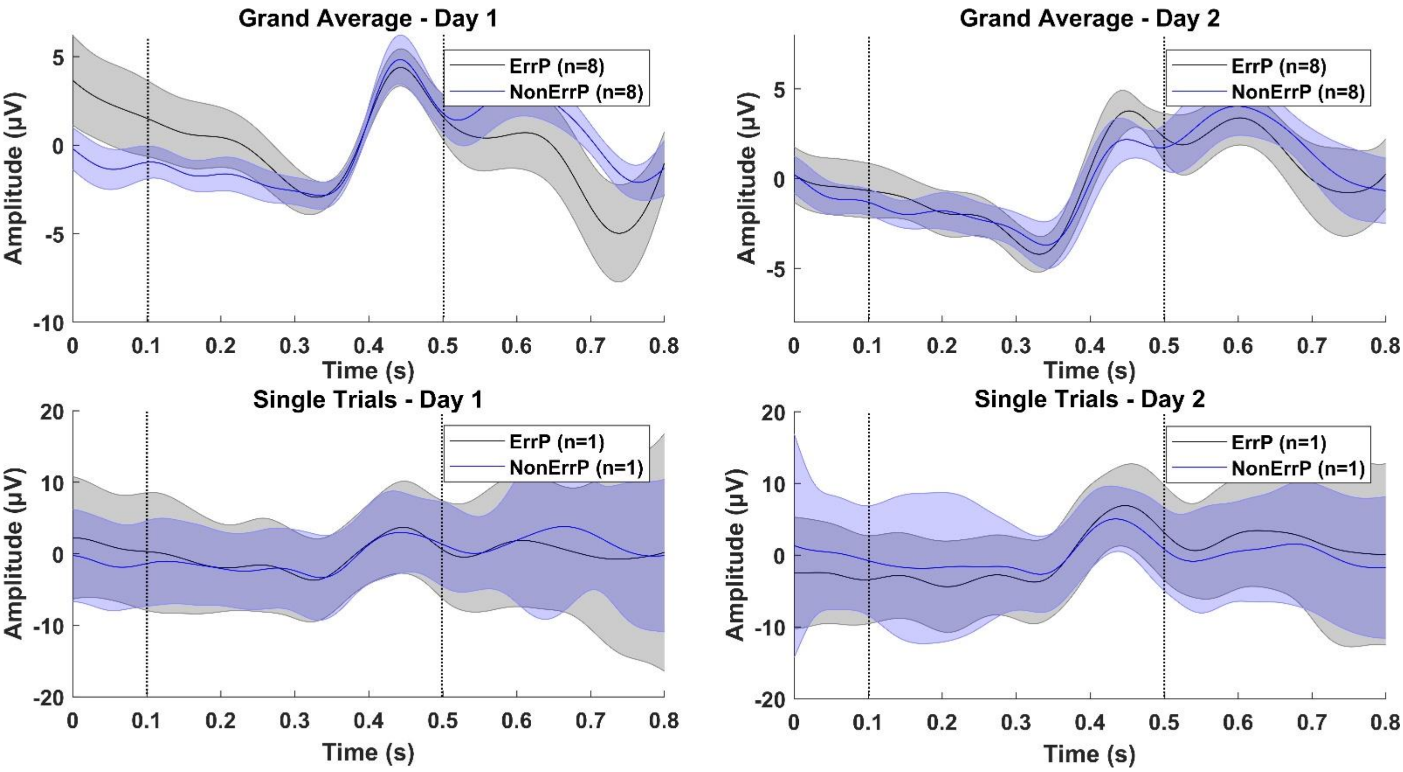

Figure S1. Top: Grand average across eight able-bodied participants for the ErrP and NonErrP epochs for recording day 1 and 2. The shaded area indicates the standard error across participants and the solid line is the mean. Bottom: Single trials for participant 1 for recording day 1 and 2. The shaded area indicates the standard deviation and the solid line is the mean across the trials. Time '0 s' is the onset of the presentation of the feedback. The signals in all trials are from the electrode position FCz. The vertical dotted lines indicate the part of the signal that was used for the classification analyses.
.

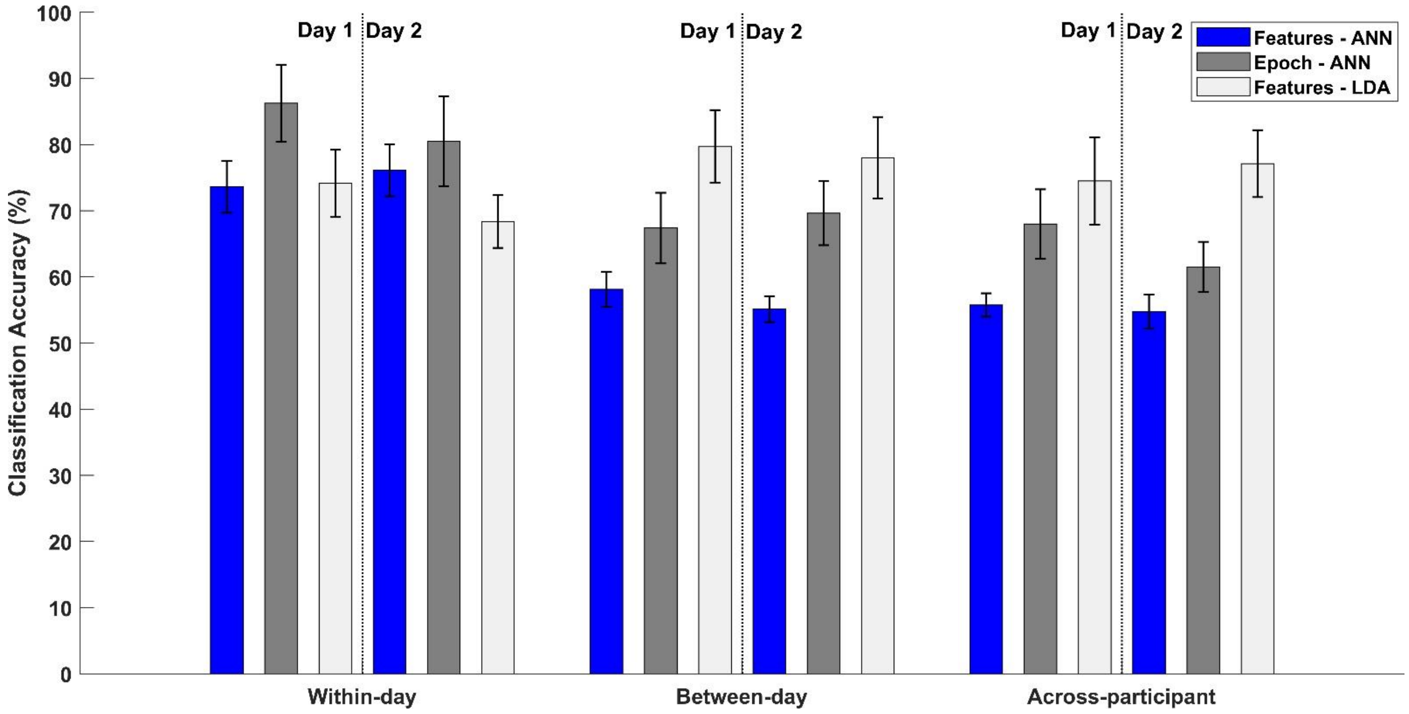

Figure S2: Classification accuracies associated with different calibration schemes using features or the entire epoch as input for the artificial neural network and classification of features using linear discriminant analysis. The bars represent the mean ± standard error across the able-bodied participants. For the between-day calibration, the three bars under "Day 1" represent training the classifier on data from recording day 1 and testing on recording day 2 and vice versa for the three bars under "Day 2".
